# Educational Programme for Cancer Nurses in Genetics, Health Behaviors and Cancer Prevention: A Multidisciplinary Consensus Study

**DOI:** 10.3390/jpm12071104

**Published:** 2022-07-05

**Authors:** Celia Diez de los Rios de la Serna, Paz Fernández-Ortega, Teresa Lluch-Canut

**Affiliations:** School of Nursing, Faculty of Medicine and Health Sciences, Bellvitge Campus, Barcelona University, 08036 Barcelona, Spain; mfo@iconcologia.net (P.F.-O.); tlluch@ub.edu (T.L.-C.)

**Keywords:** hereditary cancer, hereditary cancer syndromes, genetic testing, nurse education, health behaviors, behavioural change

## Abstract

(1) Background: Most common hereditary cancers in Europe have been associated with lifestyle behaviors, and people affected are lacking follow up care. However, access to education programmes to increase knowledge on cancer and genetics and promote healthy lifestyle behaviors in people at high risk of cancer is scarce. This affects the quality of care of people with a hereditary risk of cancer. This study aimed to reach a multidisciplinary consensus on topics and competencies and competencies that cancer nurses need in relation to cancer, genetics, and health promotion. (2) Methods: A two-round online Delphi study was undertaken. Experts in cancer and genetics were asked to assess the relevance of eighteen items and to suggest additional terms. Consensus was defined as an overall agreement of at least 75%. (3) Results: A total of 74 multiprofessional experts from all around the world participated in this study including healthcare professionals working in genetics (39%), researchers in cancer and genetics (31%) and healthcare professionals with cancer patients (30%). Thirteen additional items were proposed. A total of thirty-one items reached consensus. (4) Conclusions: This multidisciplinary consensus study provide the essential elements to build an educational programme to increase cancer nurses’ skills to support the complex care of people living with a higher risk of cancer including addressing lifestyle behaviors. All professionals highlighted the importance of cancer nurses increasing their skills in cancer and genetics.

## 1. Introduction

Cancer is the result of genetic changes in the cells that affect cell function [1]. While it can affect anyone, some people are at a higher risk than others. Altogether, about 5% to 10% of cancers are attributable to hereditary genetic alterations [2]. These mutations are infrequent, affecting just over 1% of the population [3], but they considerably increase the risk of developing more than one type of cancer.

Research advances and increased knowledge are paving the way for a greater role of genetics in oncology, offering different possibilities to those with cancer due to a hereditary genetic syndrome. Personalised cancer medicine has become crucial to every stage of cancer care, from prevention to treatment. Next generation sequencing (NGS) using panels of multiple genes that could be linked to cancer risk and the increasingly affordable prices of genetic testing have fostered these changes.

Different authors have investigated whether the population should be routinely screened for syndromes such as hereditary breast and ovarian cancer or Lynch syndrome, as the identification of these syndromes before a cancer diagnosis offers screening and prevention options [4,5]. The detection of genetic mutations also affects cancer treatment options for the individual and preventive approaches for their family members. For example, BRCA carriers can benefit from the development of targeted treatments such as PARP inhibitors [6], and subsequent to a carrier diagnosis, family testing is recommended. This offers new opportunities for early detection but poses challenges for the healthcare system and the patient in charge of family communication [7].

People suspected of having a hereditary genetic syndrome traditionally see a genetic counsellor, who helps them to understand their genetic risk and decide how to use the new information to adapt their behaviour [8]. In oncology, this activity is focused on patients and their relatives who may be at increased risk of cancer even if they do not currently have a diagnosis. This process involves medical, psychological, and family implications and should include information on therapeutic and preventive decisions along with individual, family, and behavioural risks [9].

However, the increasing role of genetics in oncological treatments poses a challenge to the healthcare system. Families with genetic syndromes expect well-trained professionals to assist in their long-term management, but they often perceive a lack of knowledge in the healthcare professionals performing their follow-up and feel frustration when looking for answers to their questions and worries [10,11]. There is a need to increase the knowledge of oncology professionals on genetics. Experts in cancer and genetics and genetic counsellors from around the world agree that healthcare systems are not prepared to absorb the rising demand for genetic testing. Most also agree that healthcare professionals not working in genetics need to have a role, but the lack of adequate knowledge poses a risk of mismanaging the results [12,13].

For the comprehensive management of people with genetic syndromes, educational needs go further than genetics [11]. Preventive health interventions for people at increased risk of cancer need to focus on health promotion and behaviour, not only on risk reduction strategies such as mastectomies or colonoscopies. Some hospitals have clinical services for people with a family history of cancer, including behavioural counselling and screening, but this is still uncommon [14,15]. Other centres are working to implement mainstream services or specialised follow-up care for families. All these services are delivered mainly by cancer nurses, who play a central role in providing information and empowering patients to take control and participate in their care [16].

However, advances in treatments and rising demands from patients are outpacing progress in training for healthcare professionals. Studies worldwide show that nurses’ knowledge about cancer and genetics and hereditary cancer syndromes is low, and they lack confidence when talking about genetics [17,18,19,20]. The International Society of Nurses in Genetics (ISONG) highlighted the need to train nurses and build their confidence in talking about genomics in order to better serve their patients [21].

Some countries and professional societies are already studying and incorporating cancer and genetic competencies for nurses, but most competency frameworks are not specific to oncology and do not include competencies on health promotion and lifestyle behaviours [21,22,23]. While health promotion is normally part of nursing training there is little involvement of cancer nurses in cancer prevention and risk reduction strategies and to empower individuals to take control and participate in their care [24]. The European Oncology Nursing Society developed the EONS Cancer Nursing Education Framework [25], which includes hereditary cancer syndromes, health promotion and risk reduction strategies in its competencies. Access to training on hereditary cancer syndromes is scarce [26] and generally focuses on assessment and testing, not health promotion and follow-up care.

The aim of this study was to reach a consensus on the topics and competencies that cancer nurses need in relation to cancer, genetics, and health promotion. These results can guide the development of a comprehensive educational programme for cancer nurses.

## 2. Materials and Methods

The study used a Delphi technique [27] to collect professionals’ perspective on training needs for cancer nurses related to the topics of genetics, hereditary cancer syndromes, and health promotion.

This investigation is part of a larger project on the role of nurses in health promotion for people with hereditary cancer syndromes. Ethics approval was obtained from the University of Barcelona’s Research Ethics Committee (IRB00003099).

### 2.1. Delphi Study Process

The Delphi method was applied to achieve consensus among a panel of experts about the interventions and knowledge needed to promote healthy behaviours in people at a high risk of cancer [28]. Delphi studies were previously used to plan educational curricula, as using expert clinicians’ opinions for this aim is appropriate [29,30]. The technique requires input from experts in the form of anonymised opinions, which are then subjected to a participatory review of the general results that informs the next round, allowing panellists to reflect and revalidate their decisions [31].

A two-round online Delphi survey was performed between February and April 2022. A third round was planned but not needed, as all items reached consensus in the second round. The institution’s Microsoft Forms was used to develop the online questionnaire and to keep the information secure on the university servers.

The first step for developing the Delphi survey drew on data from a previous systematic review by the authors of this study to identify what interventions had been administered to patients with a high risk of cancer [32]. There were also items from the module on risk reduction, early detection, and health promotion in cancer care from the EONS Cancer Nursing Education Framework [25]. Items were divided into domains on genetics, behaviours, and communication and barriers, and each included knowledge and skills competencies.

In total, the literature review yielded 18 potential topics based on skills and knowledge competencies; these were incorporated into an online survey. The first round was open for four weeks, and participants were asked to rate the importance of topics for inclusion in the programme using a seven-point Likert scale (1–3 = not important, 4 = unsure, 5–7 = important). In the first round, the questionnaire also contained free text fields where experts could suggest additional competencies not mentioned in the survey.

The survey was first piloted with two experts acquainted with the authors; after incorporating their suggestions, it was sent to the participants [33].

The first round also elicited information about the experts themselves, including country of residence, profession, years of working experience in cancer care, years of working experience in cancer and genetics, and professional roles. Their email was requested in order to contact them for the next rounds.

Data were exported to an Excel spreadsheet, anonymised by one investigator (C.D.R.S.), and analysed by all authors (C.D.R.S., P.F.-O., T.L.-C.). The authors undertook a descriptive analysis of the Likert scale, measuring the percentage of agreement about which items were important, not important, or of uncertain importance. As with the size of the panel, there is no general definition for what constitutes consensus in Delphi studies, but a threshold of 60% or more is typical; for this study, consensus was defined as agreement of 75% or more [31]. Content analysis was used to evaluate comments and suggest additional items. Items were grouped into themes and were either incorporated into existing competencies when appropriate or defined as new competencies by author agreement.

In the second round, experts had access to the previous round of results. They gave their opinion on all the first-round items as well as on the new items generated from the suggestions in the previous round. Reminders were sent to all the experts from the first round after two weeks. This round also had a final free text box for comments. All the final results and comments were shared with the participants.

### 2.2. Expert Panel

All participants were health professionals from different European countries with expertise in cancer and genetics. Invitations were sent to different members of multidisciplinary care teams with expertise in high-risk cancer patients, including oncologists, cancer nurses, genetic counsellors, nutritionists, physical therapists, psychologists, and other professionals involved in care for these patients. In addition, researchers and other people with specific expertise in the field were invited, such as authors of papers relevant to the topic and members of the European Society of Human Genetics, Cancer Genetics Group, the International Society of Nurses in Genetics, and the Global Genomics Nursing Alliance group. The study was also advertised on Twitter to identify possible participants. Other experts joined the panel through snowball sampling, as panellists were encouraged to invite other experts. Participants that expressed interest received a link to the online survey.

There is no defined number for a Delphi study panel, but as it depends on a group of experts to reach consensus, a panel of around 18 experts is recommended to ensure sufficient contributions. Assuming an attrition rate of 50%, this study planned to recruit a minimum of 36 participants [33,34].

## 3. Results

**Figure 1:**
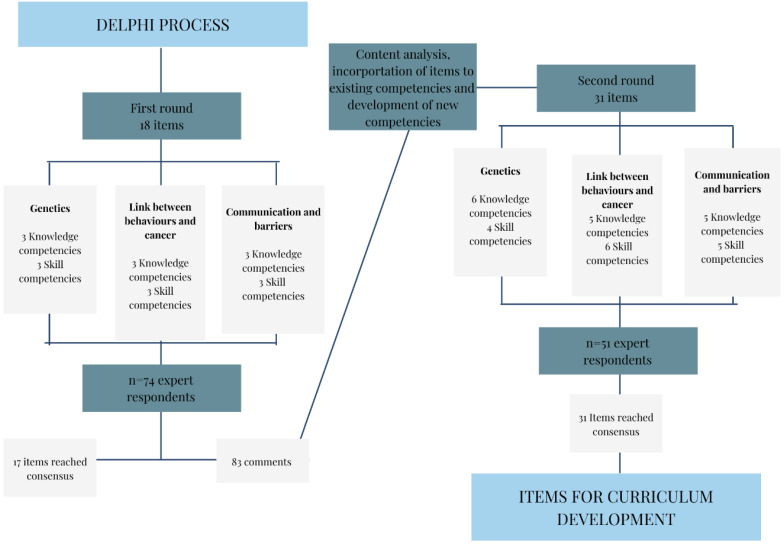
Flow chart of the Delphi process.

The Delphi study took place between January and April 2022. Details of the Delphi process can be seen in Figure 1.

### Expert Characteristics

**Figure 2:**
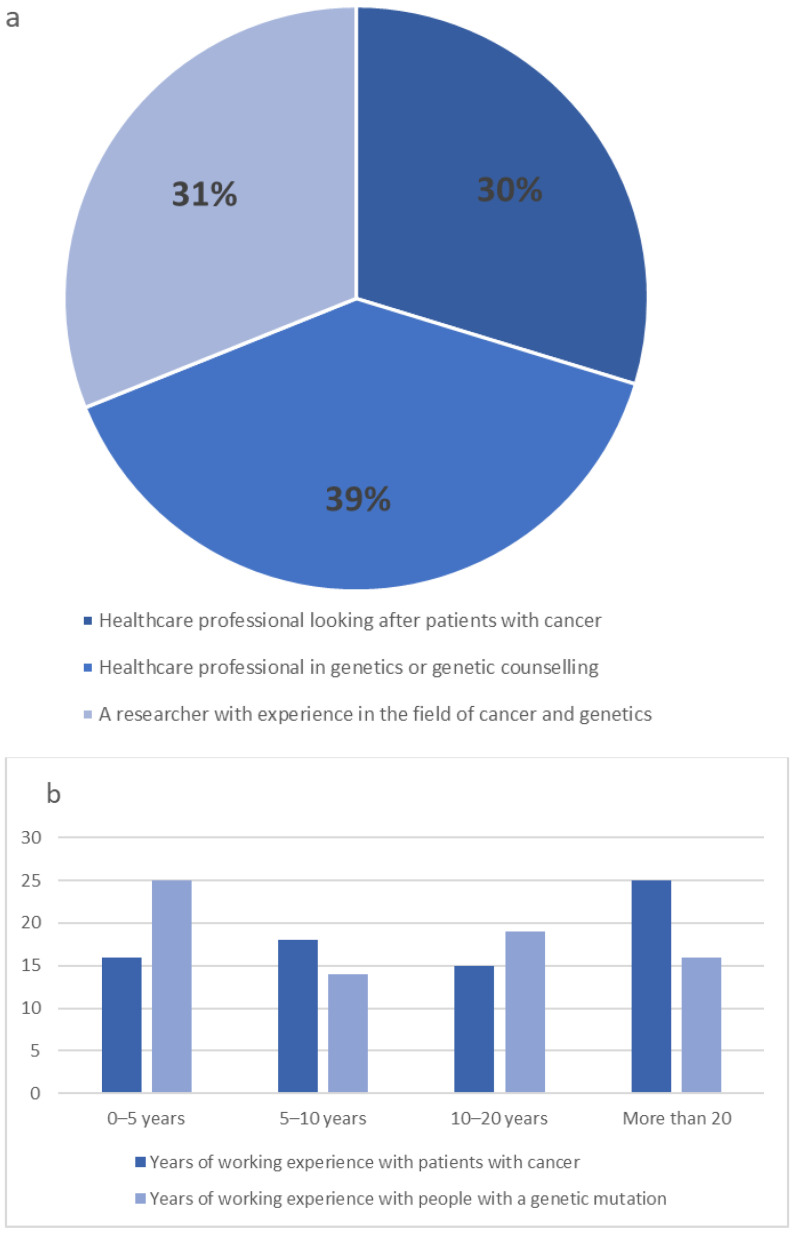
Description of the Delphi participants (**a**) Clinical experience of the Delphi participants (**b**) Years of experience of the Delphi participants.

In the first round, 74 experts participated in the Delphi study. All panellists were healthcare professionals from different backgrounds and professions with expertise in both cancer and genetics. Thirty-nine per cent were healthcare professionals working in genetics, 31% were healthcare professionals looking after cancer patients, and 30% were researchers in cancer and genetics (Figure 2a). Panellists were nurses (n = 30), physicians (n = 20), genetic counsellors (n = 9), academics/researchers (n = 10), nutritionists (n = 3), and psychologists (n = 2). Forty-four per cent had more than 20 years of experience (Figure 2b).

**Figure 3:**
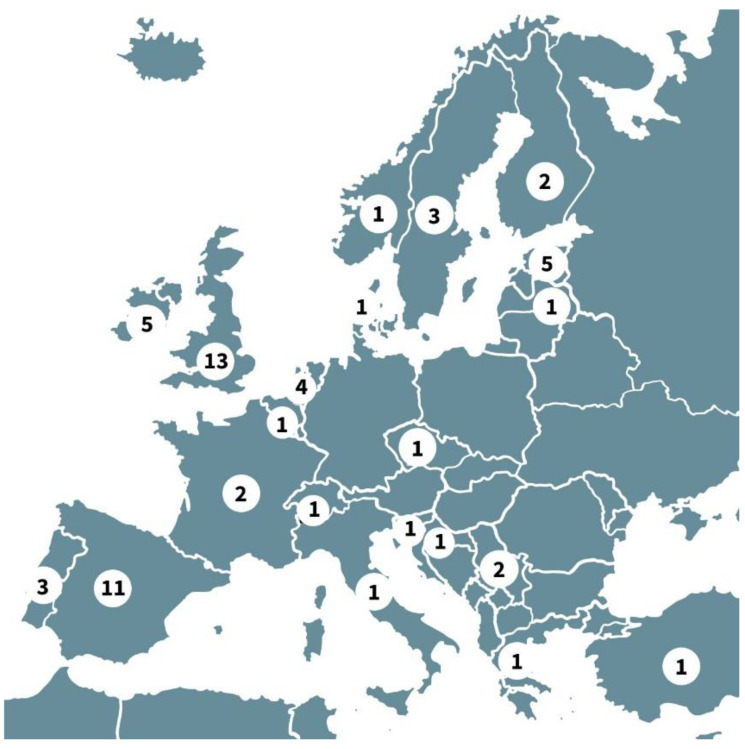
Map of European participants n = number of participants from each European country.

Participants were predominantly from Europe (n = 61), but there were also three participants from Hong Kong, five from the USA, two from Australia, one from Israel, one from New Zealand and one from Japan. Of the 82.4% of European experts, most were from the UK (n = 13) and Spain (n = 11). There were also participants from Estonia (n = 5), Ireland (n = 5), the Netherlands (n = 4), Sweden (n = 3), Portugal (n = 3), Finland (n = 2), France (n = 2), and Serbia (n = 2), as well as one each from Belgium, Croatia, Czechia, Denmark, Greece, Italy, Latvia, Norway, Slovenia, Switzerland, and Turkey (Figure 3).

In the first round, the panel reached a consensus on 17 of the 18 items proposed being classified as important. The remaining competency, “Knowledge on the European Code Against Cancer recommendations”, was important for just 68% of the panellists, and some people reported not being aware of the code or thinking there could be many other recommendations, such as the World Cancer Research Fund recommendations. Experts also provided 87 comments that were subsequently analysed.

Some of the suggestions were similar and therefore grouped together; for example, three panellists suggested skills for drawing pedigrees; five on basic knowledge on counselling; and four on competencies for follow-up and support after diagnosis with genetic counselling. The 87 comments were consolidated into 42 items. Then, those that could be encompassed under existing competencies were added with an explicit specification so participants in round 2 could see how their comments were used to modify existing items or create new ones.

The second round listed 31 items in the following two groups: existing items where participants could see results from round 1 and new items based on panellist proposals. The round 2 survey also had an option to provide free text comments at the end.

Fifty-one (68.9%) participants from the first round completed the round 2 survey. All the items were considered important by enough participants to reach consensus. The degree of consensus was 80% or higher for most items, while only the following two items had a lower level of agreement, at 76%: “Knowledge of recommendations to reduce risk of cancer (the item previously called “Knowledge on the European Code Against Cancer recommendations”) and “Knowledge on health belief theories and health behaviour change theories”, which yielded a 78% consensus during the first round (Table 1).

There were also 16 comments with recommendations for future education. As there were many topics for training, experts recommended dividing the training into three themes (genetics, behaviour, and communication) or into different levels of training (essential, intermediate, and advanced training). Others commented on the delivery methods (proposing case studies and online learning). The results and a summary of the comments were sent to the participants.

## 4. Discussion

This study served to identify the competencies that cancer nurses should obtain in cancer genetics and cancer prevention. The main competencies were defined by a Delphi panel of international experts in cancer and genetics. This is the first time that a study aimed to develop a consensus on the competencies needed in cancer, genetics and prevention, and communication barriers. The multidisciplinary panel rapidly reached a high level of consensus, with all participants recognising the importance of developing oncology nurses’ competencies in these fields.

There have been studies focused on creating competencies in cancer and genomics for healthcare professionals, but most were not specific to nurses and did not incorporate individual risk reduction behaviours and health promotion skills. A recent study developed competencies of healthcare professionals (including nurses) in cancer genomics [35]. This study focused on knowledge, attitudes, and skills needed for nurses and physicians, identifying 42 items just in cancer and genomics. We looked at the items described in that study and found that all necessary items were reflected in the competencies of the present study. In that study, the only item for prevention was the “Ability to use health promotion/disease prevention practices that incorporate genetic and genomic information as well as personal and environmental risk factors” in brackets; however, the authors focused more on preventive management than personal behaviours, adding “giving advice and discussing preventive management such as mammography, colonoscopy”. Their Delphi panel was also limited to a small sample of six geneticists and doctors, failing to include nurses in a study that aimed to develop nursing competencies [35]. Cancer nurses require education tailored to their needs and competencies, especially when it comes to their role in following up with these individuals after diagnosis [20,26].

People with a genetic predisposition to cancer see different healthcare professionals but generally have little engagement in health promotion interventions, as their knowledge of cancer predisposition syndromes is limited [36]. There is a need to develop more interventions for these patients and measure multiple outcomes, including health behaviours and changes as well as the person’s wellbeing. Cancer prevention and education to improve health literacy is important for the population but even more so for those who are already worried about their cancer risk [37]. To make this possible, healthcare professionals have to improve their own knowledge on cancer genetics, prevention strategies, and behaviours that affect cancer risk, but they also need to understand and support behavioural change [38]. Recent studies show how these patients generally mistrust the information they receive from healthcare professionals about their genetic syndrome and about prevention and screening practices, as they are not informed of what a cancer predisposition to a pathogenic variant means [36,39].

Cancer nurses are well positioned to evaluate and support these individuals and their families, and there is a need for them to assume this role to favour the mainstream implementation of genetics in oncology. A multiprofessional study examining the activities of nurses in cancer screening identified patient education and health promotion as core activities of these nurses [40]. Interestingly, physicians could envision the potential for nurses’ activities in research, patient education and evaluation of interventions more clearly than nurses themselves. The author speculated that social desirability bias could have affected this result, as physicians were reporting what nurses could provide while the nurses were describing what they were actually doing in practice.

The competencies obtained in the present study lay the foundation for the development of cancer genetics and cancer prevention training for cancer nurses. The expert feedback highlighted the importance of cancer nurses developing their knowledge, knowing the role of genetic counsellors, working together, and the involvement of experts in patient follow-up. Genetic counsellors have and will continue to play a key role in helping patients and families before and after testing as well as supporting oncology healthcare professionals in mainstreaming this model. These professionals play a crucial part in educating healthcare professionals in genetics and have unique knowledge on counselling.

### Strengths and Limitations

The main limitations of the study are related to the Delphi method. Despite being an effective tool to reach consensus and to plan educational curricula, this technique is subject to bias [29,30,35]. In this case, the competencies proposed in the survey were based on a previous systematic review. While this is very helpful, it may also limit participants’ discussion and ideas, affecting the validity by influencing the first part of the survey [31,41]. For this reason, the authors did leave room for participants to add their suggestions and encouraged their ideas and participation, which improved the second round. Moreover, the anonymity of the Delphi technique removed any group pressure, reducing the potential for spurious changes in answers [33]. Another common limitation of Delphi studies [41], which also applies to this one, is the attrition rate. Even though participants received reminder emails, and the second round was left open longer than initially planned, the attrition rate between the first and second round was 31%.

However, the study method also offers strengths. The Delphi technique is useful in that it allows for the involvement of different expert professionals from various countries. As this study is part of the exploratory phase of a larger project, this engagement enriches the project with multiprofessional and multicountry expertise [31,33]. The Delphi process and anonymity was followed rigorously. This study was also piloted before the implementation of the first round, something highly recommended for Delphi studies but not commonly followed [41].

Another strength resides in the composition of the expert panel. Apart from the relatively large sample size in both rounds, panellists derived from numerous countries (especially in Europe), and the results were strengthened by their expertise. Methodological guidance suggests Delphi studies seek similar representation from each area of expertise, and this study the panel was very evenly divided into the following three backgrounds: healthcare professionals working in genetics, healthcare professionals looking after cancer patients, and researchers in cancer and genetics [27]. It also had representation from different healthcare professionals. The high rate of agreement and similar consensus rates obtained in the two rounds suggest stability, making the results more reliable [27].

While the expert panel offered many strengths, the composition of professionals, with 82.2% from European countries, poses a limitation as the results may not represent what educational needs other countries from outside Europe may have on these topics. Though professionals from outside of Europe participated on this consensus study, experts from outside of Europe could be consulted to ensure the validity of the educational programme outside the represented countries. Future studies should also look at patients’ views on the education required for cancer nurses.

## 5. Conclusions

The introduction of genomics in oncology has changed all aspects of cancer care and provides many prevention opportunities. Healthcare professionals have demonstrated interest in involving cancer nurses, raising their awareness of the importance of genetics, and training them to incorporate cancer prevention in their daily practice.

This Delphi study aimed to achieve consensus on the competencies needed for a cancer nurse education programme in cancer genetics and cancer prevention. The results of this study, which involved experts from all around the world, show that healthcare professionals consider it important for cancer nurses to increase their knowledge and skills on these topics. This study will serve as the basis for the content of the educational programme to promote cancer and genetics and cancer prevention education for cancer nurses.

## Figures and Tables

**Table 1:** Educational items proposed in the two Delphi rounds and % of agreement.

Genetics	Round 1 Agreement	Round 2 Agreement
Knowledge on determinants of cancer	100%	100%
Knowledge and understanding of the most common genetic mutations/syndromes in cancer setting	95%	95%
Knowledge of the role of genetics in cancer treatment	93%	95%
Knowledge of instruments to estimate risk	NEW in 2	80%
Knowledge on genetic processes	NEW in 2	88%
Knowledge of the role of genetic counsellors	NEW in 2	80%
Ability to undertake a comprehensive history to identify the individual, familial, genetic, sociocultural, economic and environmental factors	93%	96%
Ability to identify individuals that may be potentially at risk of having a genetic predisposition to cancer	97%	96%
Ability to create communication links between oncology and genetic healthcare providers	96%	92%
Ability to explain patients genetic testing	NEW in 2	84%
**Behaviors**	**Round 1**	**Round 2**
Knowledge on modifiable determinants of cancer and their importance on people with high risk of cancer	92%	90%
Health promotion and health education	91%	84%
Knowledge on the European Code Against Cancer recommendations changed in the second round to: Knowledge of recommendations to reduce risk of cancer	64%	76%
Knowledge of the social and behavioural determinants of health on genetic susceptibility	NEW in 2	86%
Surveillance	NEW in 2	88%
Ability to use health promotion/disease prevention practices that incorporate genetic and genomic information as well as personal and environmental risk factors	91%	88%
Ability to address peoples’ beliefs and values	92%	94%
Ability to identify problems with surveillance	86%	92%
Ability to recognise risk factors	NEW in 2	80%
**Communication**	**Round 1**	**Round 2**
Barriers to effective information provision	92%	93%
Awareness of consequences of cancer such as the emotional experiences associated with the diagnosis of cancer, the impact on the life of the patient and family as well as effects of treatment	97%	100%
Knowledge on health belief theories and health behaviour change theories.	78%	76%
Family planning and fertility implications	NEW in 2	90%
Psycho-social support	NEW in 2	88%
Ability to identify ethical, ethnic/ancestral, cultural, religious, legal, fiscal, and societal issues related to understanding health and genetic information	77%	86%
Demonstrate use of a range of effective communication skills/strategies to provide information, psychological and emotional support to individuals and communities about cancer	96%	96%
Select and adopt an appropriate communication approach, from a range of core communication and consultation skills, to effectively support the people with high risk of cancer	95%	96%
Ability to communicate and support family members at risk	NEW in 2	92%
Nurses’ role in the follow up/support	NEW in 2	92%

## Data Availability

Not applicable.
